# Analyzing the importance of attributes for Brazilian consumers to replace conventional beef with cultured meat

**DOI:** 10.1371/journal.pone.0251432

**Published:** 2021-05-07

**Authors:** Gabriela Andrade de Oliveira, Carla Heloisa de Faria Domingues, João Augusto Rossi Borges

**Affiliations:** Federal University of Grande Dourados, Dourados, Mato Grosso do Sul, Brazil; Tokat Gaziosmanpasa Universitesi, TURKEY

## Abstract

Cultured meat has been proposed as an alternative source of protein to overcome the environmental and ethical problems associated with conventional meat production. However, the lack of consumers’ acceptance could be a major barrier to the introduction of cultured meat on a large scale. Despite Brazil being one of the countries that consumes the most meat per capita, little is known about Brazilian consumers’ preferences for alternative meat. The objective of this study is to identify which attributes influence consumers to possibly replace conventional beef meat with cultured meat in Brazil. An online survey was conducted, and Best-worst scaling methodology was applied to a sample of 225 consumers. The sampling leaned towards educated and employed residents of the southeast region of Brazil, which might not fully represent the Brazilian population. Despite limitations in terms of the sampling demographic, overall, Brazilians appear to be willing to consume cultured meat: 80.9% of the sample would be willing to try it, 61.3% would be willing to eat it regularly, and 56.9% would be willing to eat cultured meat as a replacement for conventionally produced beef. Despite the focus of this study being on attributes of a hypothetical product that is not commercially available, which might pose difficulty to consumers to predict their future consumption behavior, results show that the most important attributes influencing consumers to possibly replace conventional beef meat by cultured meat in Brazil are anticipated risk of zoonotic diseases, anticipated healthiness and anticipated food safety conditions. Attributes related to benefits at a global societal level and intrinsic characteristics of cultured meat were less important.

## Introduction

Cultured meat has been proposed as an alternative source of protein to overcome the environmental and ethical problems associated with conventional meat production [1]. However, the lack of consumers’ acceptance could be a major barrier to the introduction of cultured meat on a large scale [2, 3]. Around the world, scholars are now interested in understanding how consumers will react to cultured meat. Most of the research to date has been conducted with consumers in the US and in European countries. Evidence suggests that consumers’ preferences for cultured meat vary across countries and cultures, and therefore expanding the research elsewhere in the world is warranted [1, 4, 5]. Such studies are important to inform future marketing or regulatory strategies [1].

Despite Brazil being one of the countries that consumes the most meat per capita (around 97 kg per capita per year), little is known about Brazilian consumers’ preferences for alternative meat (with the exception of the studies [6, 7]). Therefore, there is a need to develop a deeper understanding of how Brazilian consumers will react to cultured meat.

Several approaches have been used to understand how consumers will react to cultured meat. A common approach is to conduct online surveys to investigate consumers’ perceptions and attitudes toward cultured meat, and their willingness to try, buy or eat it. In a study conducted in the US, Wilks and Phillips [8] found that the main concerns associated with cultured meat were anticipated price, limited taste and appeal, and a concern that cultured meat was not natural. In another online survey conducted in the US, Wilks et al. [9] found that food neophobia, disgust sensitivity, distrust in food scientists, and conspiratorial ideation were associated with negative consumers’ reactions to cultured meat. Mancini and Antonioli [5] conducted an online survey with Italian consumers and their results showed that participants were more positive about externalities (e.g., sustainability, animal welfare, and security) than intrinsic (e.g., safety, taste, and nutrients) characteristics of cultured meat. Weinrich et al. [10], in an online survey conducted with German consumers found that ethical issues (e.g., animal welfare, ecological) were associated with positive reactions to cultured meat, whereas concerns of unnaturalness, healthiness, taste and adverse consequences for traditional farmers were associated with negative reactions. Verbeke et al. [11] conducted focus group research and an online survey with consumers from Belgium, Portugal, and the United Kingdom. Participants initially expressed disgust and judged cultured meat as unnatural. Few participants associated the consumption of cultured meat with personal benefits (e.g., taste, personal health), but instead they associated consumption of cultured meat with benefits for the society, mainly environmental and ethical ones. Participants also associated consumption of cultured meat with risks to human health, to adverse societal consequences (e.g., loss of farming traditions and agricultural job), and to concerns about risk governance and control. Bryant et al. [4] conducted online surveys in US, India and China. In the three countries, higher familiarity was associated with higher acceptance of cultured meat. In a combination of online and paper-based surveys, Gómez-Luciano et al. [6] investigated consumers’ willingness to purchase cultured meat in the United Kingdom, Spain, Brazil and the Dominican Republic. They found that beliefs that cultured meat is healthy, safe, and nutritional were associated with higher willingness to purchase cultured meat in Brazil. In an online survey conducted with highly educated residents of two cities in the south of Brazil, Valente et al. [7] found that few participants had knowledge about cultured meat, and that participants associated animal welfare and environmental conditions as potential benefits of consumption of cultured meat whereas the associated harms were economic (likely, concerns about price) and health.

Another approach to conduct survey-based assessments is to use experimental approaches. In this strand of literature, Bekker et al. [12] used Solomon four-group experiment design and found that provision of information about the sustainability of cultured meat was associated with consumers having more positive attitudes towards cultured meat. Siegrist et al. [3] conducted online experiments to examine the impact of perceived naturalness and disgust on consumer acceptance of cultured meat. They found that a low level of acceptance was associated with perceptions that cultured meat is unnatural, and that how cultured meat was described to participants influenced acceptance. In a hypothetical choice experiment, Slade [13] investigated consumers’ willingness to purchase burgers made from cultured meat. Results showed that demographics such as age, gender, and attitudes towards the environment and agriculture were associated with willingness to purchase. Bryant and Barnett [14] used an online experimental between-subjects design to investigate the effect of different names for cultured meat on consumer’s acceptance. Their results confirmed that how cultured meat is named impacted consumer acceptance.

In this study, the objective was to identify which attributes influence consumers to possibly replace conventional beef meat with cultured meat in Brazil. To identify the attributes, Best-worst scaling (BWS) methodology was applied, which is a novel approach to understand consumers’ preferences for cultured meat. BWS provides some advantages compared to the Likert-based scales commonly used to elucidate consumers’ preferences. Advantages of BWS include task comprehension (i.e., relatively easy for most participants to understand), and free of scale-use bias (i.e., when participants use only part of the scale or when participants need to map their preferences onto the scale in the same way) [15, 16]. It is hoped that this study will not only expand the understanding of how Brazilian consumers will react to cultured meat, but that it will also be relevant to a broad audience as it identifies the most and least important attributes influencing consumers to possibly replace conventional beef meat with cultured meat.

## Materials and methods

### Sampling and data collection

An online survey was conducted among Brazilian consumers from June 2019 to July 2019. The questionnaire was supplied by a company specialized in market research. We are aware of the limitation that online representativeness does not fully equal representativeness of the whole Brazilian population. Therefore, our results must be viewed with care. The margin of error is 7%.

The survey questionnaire was divided in three sections. The first section included the demographics age (18–29 years, 30–49 years, > 50 years), gender (female or male), educational attainment (no formal schooling, incomplete elementary school, complete elementary school, incomplete high school, complete high school, incomplete bachelor degree, complete bachelor degree, postgraduate studies), monthly income (no income, up to R$998,99, from R$998,99 to R$2.996,97, from R$2.996,97 to R$5.993,94, from R$5.993,94 to R$8.990,91, above R$8.990,91), employment (unemployed, student, employed, entrepreneur, retired, housewife, others), and living region in Brazil (South, Southeast, Center west, Northeast, North).

The second section of the questionnaire included the food consumption patterns: dietary lifestyle (meat consumers, vegetarian, vegan, others) and weekly beef consumption (no consumption, 1–2 meals, 3–5 meals, 6–10 meals, more than 10 meals). In this section, consumers were asked about perceived naturalness of cultured meat compared to conventional beef meat (scale from 1 to 5, where 1 much less natural and 5 much more natural), their willingness to try cultured meat (yes or no), willingness to eat cultured meat regularly (yes or no), and willingness to eat cultured meat as a replacement for conventionally produced beef (yes or no).

The third section of the questionnaire used the Best-worst scaling methodology focused on cultured meat attributes. This part of the questionnaire is explained in the next section. Before data collection, the questionnaire was pre-tested with 15 consumers, but no substantial changes were necessary. All the questions were translated to Portuguese. Survey questions in Portuguese are presented in S1 Survey. To ensure correct translation, the questions were translated back to English. Survey questions in English are presented in S2 Survey. This project received research ethics board approval from Federal University of Grande Dourados/Faculty of Management, Accounting and Economics. A written informed consent was obtained from each participant prior to participating in this research.

### Best-worst scaling and cultured meat attributes

In applications of the best-worst scaling, participants choose among a large set of attributes of a specific product (e.g., cultured meat) the one that they like the most and the least. There are many attributes that influence consumers’ preferences for cultured meat. Based on the literature review presented in the Introduction, ten attributes were chosen to represent the range of choice cues that consumers might use to replace conventional beef meat with cultured meat. The list of selected attributes is presented in Table 1.

**Table 1:** List of attributes used for the best-worst analysis.

Attributes	Presentation of the attributes in the questionnaire
Anticipated food safety conditions	If cultured meat was safer for consumption than conventional beef meat.
Anticipated healthiness	If cultured meat were heathier than conventional beef meat.
Anticipated risk of zoonotic diseases	If cultured meat were less risky for zoonotic diseases than conventional beef meat.
Anticipated societal impacts	If cultured meat had no negative impacts on traditional farming.
Anticipated environmental impacts	If cultured meat caused less harm to the environment than conventional beef meat.
Anticipated animal welfare conditions	If cultured meat were more animal friendly than conventional beef meat.
Anticipated taste	If cultured meat were tastier than conventional beef meat.
Anticipated appearance	If cultured meat had a superior appearance than conventional beef meat.
Anticipated popularity	If cultured meat were more popular than conventional beef meat.
Anticipated price	If cultured meat were cheaper than conventional beef meat.

These attributes were presented in the questionnaire in a choice set. In each choice set, participants were presented with four attributes, and they were asked to choose the best and the worst attribute. Each attribute was presented to participants four times. Each participant faced ten choice sets. The selection of ten choice sets satisfies the design characteristics of one and two-dimensional frequency balance, positional balance, and connectivity [17]. The experimental design of the questionnaire was constructed using the MaxDiff Sawtooth Software module. To minimize bias order, 250 different versions of the questionnaire were used.

To ensure that all participants possess the same basic information about cultured meat, they were asked to read the following text before starting to choose the best and worst attributes: “Cultured meat is meat that is grown from stem cells using tissue-engineering techniques. These cells are then transported to a food industry lab where the cells will proliferate in a nutrient-rich medium. Animals are not killed. This could be an alternative to traditional meat as we know it nowadays. Cultured meat should not be confused with meat substitutes like tofu or quorn, because it is real meat. Currently it is not commercially available, though research is being conducted to introduce it as a potential new meat production technique for the future. The unveiling of the world’s first in vitro hamburger occurred in London, August 2013. Now, imagine that cultured meat is available at supermarkets, butcher shops and restaurants. For each of the next ten choice sets, choose the most and the least important attribute that would influence you to replace conventional beef meat with cultured meat”.

### Data analyses

Descriptive statistics was used to characterize participants and their food consumption patterns. Hierarchical Bayes (HB) analyses was used to estimate scores for each participant. To facilitate interpretation, the raw scores were rescaled from 0 to 100, and the average for each attribute was reported. The higher the score, the more important the attribute. In addition, an attribute with an average of 10 is twice as important as an attribute with an average of 5. To ensure only responses of consumers who answered the questions carefully were included in the analyses, participants Root likelihood (RHL) below 0.312 were omitted from the analyses as a fit statistic below this score would suggest random answers to the choice tasks [17]. A total of 264 participants completed the questionnaire, but 39 were excluded because low RHL. The final sample comprised of 225 participants. Data is presented in S1 Raw data.

## Results and discussion

### Demographics and food consumption patterns of the sample

The demographics and food consumption patterns of the 225 participants are reported in Table 2. Nearly two-thirds of participants were younger than fifty years old. The sample was balanced in terms of gender. Forty-three percent of participants had at least completed a bachelor’s degree. The sample was dominated by participants living in the Southeast region and by people employed. Half of the sample earn a monthly income above R$2.996,97. The sample was also dominated by meat consumers. Sixty-four percent of participants consume beef meat in at least three of their weekly meals. Nearly sixty percent of participants perceived cultured meat much less natural than conventional beef meat (they answered 1 or 2 in a scale from 1 to 5, where 1 much less natural and 5 much more natural).

**Table 2:** Demographics and food consumption patterns.

Variable	Cases	%
Age	18–29 years	28.0
	30–49 years	43.6
	> 50 years	28.4
Gender	Male	46.7
	Female	53.3
Educational attainment	Incomplete elementary school	0.9
	Complete elementary school	4.0
	Incomplete high school	4.4
	Complete high school	29.8
	Incomplete bachelor degree	17.8
	Complete bachelor degree	34.2
	Postgraduate studies	8.9
Living region	South	17.3
	Southeast	54.2
	Center west	8.0
	Northeast	15.6
	North	4.9
Monthly income	No income	7.6
	Up to R$998,99	12.0
	From R$998,99 to R$2.996,97	31.1
	From R$2.996,97 to R$5.993,94	20.4
	From R$5.993,94 to R$8.990,91	15.1
	Above R$8.990,91	13.8
Employment	Student	5.3
	Employed	52.9
	Entrepreneur	11.6
	Retired	5.3
	Unemployed	7.6
	Housewife	8.9
	Other	8.4
Dietary lifestyle	Meat consumers	94.2
	Vegetarians	1.3
	Vegans	2.2
	Others	2.2
Weekly beef consumption	No consumption	4.4
	1–2 meals	32.0
	3–5 meals	39.6
	6–10 meals	18.7
	More than 10 meals	5.3
Perceived naturalness of cultured meat	1	24.4
	2	33.3
	3	31.1
	4	7.6
	5	3.6
Willingness to try cultured meat	Yes	80.9
	No	19.1
Willingness to eat cultured meat regularly	Yes	61.3
	No	38.7
Willingness to eat cultured meat as a replacement for conventionally produced beef	Yes	56.9
	No	43.1

Overall, Brazilians appear to be willing to consume cultured meat: 80.9% of the sample would be willing to try it, 61.3% would be willing to eat it regularly, and 56.9% would be willing to eat cultured meat as a replacement for conventionally produced beef. Similar studies were conducted in other countries to investigate the rate of personal willingness to consume cultured meat, each with different findings. In the US, Wilks and Phillips [8] found that 65.3% would be willing to try cultured meat, 32.2% would be willing to eat it regularly, and 31.2% would be willing to eat cultured meat as a replacement for farmed meat. In Germany, Weinrich et al. [10] reported that 57% would be willing to try cultured meat, and 30% would be willing to eat it regularly. In Italy, Mancini and Antonioli [5] found that 54.5% would be willing to try cultured meat. To sum up, the proportion of the sample willing to consume cultured meat is higher than in any previous studies. Nevertheless, the different findings reported in these studies might be explained by differences in the samples, descriptions of cultured meat, question design [1] or even the different names used for cultured meat (e.g., when the term clean meat is used, consumers tend to have more positive reactions to cultured meat than when the term lab grown meat is used) [14].

Participants were not specifically asked about their willingness to buy, purchase or pay for cultured meat. It is interesting to note, however, that Gómez-Luciano et al. [6] found that only 11.5% of consumers in Brazil would be willing to purchase cultured meat. Their assessment of willingness to purchase was formulated as “Would you personally be willing to purchase cultured meat?”, and participants answered this question in a five-point scale ranging from totally disagree to totally agree. They further analyzed the willingness to purchase by specifying the response categories “totally agree” and “agree” as “yes” and the other responses categories as “no”. In contrast to our results, they found that few Brazilians would be willing to consume cultured meat. We speculate that these different findings are underpinned by the questions used to measure personal willingness to consume cultured meat. While we measured willingness to try cultured meat and willingness to eat it regularly, Gómez-Luciano et al. [6] measured willingness to purchase it, which seem to require a fuller engagement of consumers with cultured meat.

### Hierarchical Bayes (HB) analyses

Results of the HB analyses are presented in Table 3. Results show that anticipated risk of zoonotic diseases (average 17.9) and anticipated food safety conditions (average 14.1) were the first and the third most important attributes, respectively. These two attributes were, for instance, as much as twice to three times as important as the intrinsic attribute of anticipated taste (average 7.8), and they were as much as twice to three times as important as anticipated price (average 5.5). The higher importance of a safe consumption might be explained by Grunert’s [18] argument that, under normal circumstances, safety is not an important attribute, but when consumers perceive a risk, such as when a novel product is offered, it becomes the most important attribute. The safe consumption of cultured meat was reported in previous studies as a consumer’ concern [11, 19]. Bryant and Barnett [1] argue that this concern is linked to perceptions of unnaturalness and to a sense of scientific uncertainty.

**Table 3:** Results of the Hierarchical Bayes (HB) analyses after rescaling the raw scores from 0 to 100.

Attributes	Average score after rescaling	95% Lower	95% Upper	Std. dev.
Anticipated risk of zoonotic diseases	17.9	16.9	18.9	7.3
Anticipated healthiness	17.7	17.1	18.4	4.8
Anticipated food safety conditions	14.1	13.4	14.9	5.9
Anticipated environmental impacts	12.4	11.5	13.4	7.6
Anticipated animal welfare conditions	11.7	10.8	12.6	6.8
Anticipated societal impacts	8.3	7.3	9.2	7.1
Anticipated taste	7.8	6.9	8.6	6.4
Anticipated price	5.5	4.6	6.4	7.0
Anticipated appearance	2.7	2.2	3.3	4.2
Anticipated popularity	1.8	1.4	2.3	3.2
Root likelihood				0.50^a^

^a^ The Root Likelihood measures the goodness of fit; four attributes in each choice set result in a chance probability of 0.25 for each attribute to be chosen (i.e., 1/4); the model, however, gained 0.50 correct predictions.

Anticipated healthiness (average 17.7) was the second most important attribute. The healthiness of cultured meat was also a consumer’ concern observed in previous studies [7, 8, 11] and a higher perceived healthiness has been associated with higher consumer acceptance of cultured meat [4, 6]. It was two times as important as anticipated taste (average 7.8), and it was three times as important as anticipated price (average 5.5).

Results of the HB analyses also show that next to a safe and healthy consumption, attributes related to externalities (i.e., anticipated environmental impacts, anticipated animal welfare conditions, and anticipated societal impacts) were important in influencing consumers to possibly replace conventional beef meat with cultured meat in Brazil. Previous studies found that consumers associate environmental impacts and animal welfare as benefits of consumption of cultured meat [4, 5, 7, 8, 10, 11]. Interestingly, these attributes seem related to benefits at a global societal level rather than to an individual level [11]. Other studies also observed that consumers are worried about adverse societal consequence, such as negative impacts on traditional farming [8, 11].

Results of HB analyses also suggest that anticipated price, intrinsic characteristics of cultured meat (i.e., anticipated taste and anticipated appearance), and anticipated popularity were less important in influencing consumers to possibly replace conventional beef meat with cultured meat in Brazil. Previous literature found that the lack of sensory appeal is an important reason underpinning rejection of cultured meat [8, 11, 13, 20]. Previous studies also found that anticipated price is a relevant attribute influencing consumers’ acceptance of cultured meat [8, 11, 13, 20].

## Conclusion

This study investigates the relative importance of the attributes influencing consumers to possibly replace conventional beef meat with cultured meat in Brazil. Results suggest that anticipated risk of zoonotic diseases, anticipated healthiness, and anticipated food safety conditions were the most important attributes, followed by anticipated environmental impacts, anticipated animal welfare conditions, and anticipated societal impacts. Attributes related to intrinsic characteristics of cultured meat were less important.

Our results can inform marketing campaigns aimed to increase cultured meat acceptance. Marketers must take any opportunity to inform consumers about the safety (i.e., risk of zoonotic disease and food safety) and health benefits of cultured meat. For example, messages may highlight that cultured meat contains reduced pathogens and contaminants, and it is low fat and nutritious. A previous study found that messages focusing on safety and health benefits of cultured meat are likely to be more persuasive than those focused on quality and taste [21]. Other messages may highlight the benefits of cultured meat to the environment and to animals, particularly when compared to conventionally produced beef. Several recent studies have demonstrated that cultured meat acceptance can be increased by providing additional positive information [5, 21, 22]. When commercially available, the benefits of cultured meat could be communicated to consumers, e.g., in-store, on labels, and via social media. Assuring a proper regulation to dismiss consumers’ concerns about food safety seem also crucial to drive acceptance of cultured meat. Although the relative importance of intrinsic attributes of cultured meat and anticipated prices was not high, we concurred with Verbeke et al. [11] that consumers will not be willing to compromise on these attributes. Possibly, a more realistic scenario is where cultured meat and conventional beef meat are safe to consume, have similar price, and have similar intrinsic attributes. In this case, consumers would most likely prefer cultured meat because its benefits to health, environment and to animals. Nevertheless, it is challenging for food engineers to develop cultured meat that satisfy the performance of all attributes of this study.

Cultured meat provides an attractive substitute for conventionally produced meat [22, 23]. While uncertainty ranges are large, previous studies indicated that cultured meat requires smaller quantities of agricultural inputs, water, and land than conventional meat [24, 25]. Cultured meat also presents advantages in terms of animal welfare and it is safer to consume compared to conventional meat [23, 26]. These are all benefits that society might experience if cultured meat become a food commodity. However, it is unclear its impact on rural economies [23]. Brazil is a major producer of livestock and a shift towards cultured meat might harm the Brazilian economy. This is an important drawback that futures studies might investigate.

This study has some limitations that should be considered in future research. First, this study focused on attributes of a hypothetical product that is not commercially available, and it is possible that respondents faced difficulty to predict which attributes will influence their future consumption behavior. Results indicate that perceived price and intrinsic attributes (i.e., taste and appearance) were less important than attributes related to benefits to the environment, animals and society. This might have occurred because of social desirability. This is a common limitation in studies that investigate consumers’ preferences for cultured meat [4–6, 10]. Future studies might use an experimental design and a real cultured meat product to investigate the relative importance of these attributes. In this regard, researchers might be interested to understand the form of meat that is preferred to be replaced with cultured meat (e.g. burger, stew, steak, etc.). Second, the attributes used in this study focused mostly on the advantages of cultured meat over conventional beef meat. Future studies might include potential disadvantages, such as perceived naturalness and disgust sensitivity, which are associated with negative consumers’ reactions to cultured meat [9]. Third, compared to the Brazilian population, the sample was more educated [27]. Hence, the features of the sample probably include several sources of variation that limit the generalizability of the results. In future studies, the use of random sampling procedures is recommended. This would improve the representativeness of the sample. Fourth, the survey was not designed to assess the impact of demographics on some measure of willigness to consume cultured meat. Future studies might use robust measures of willingness to consume and test who is mostly likely to consume this product. For instance, previous research has indicated that cultured meat would likely be more appealing to the younger generation [1, 8, 13].

## Supporting information

S1 Survey Survey questions in Portuguese. (DOCX)

S2 Survey Survey questions in English. (DOCX)

S1 Raw data (XLSX)

## References

[1] Bryant C, Barnett J. Consumer acceptance of cultured meat: A systematic review. Meat Sci. 2018; 143: 8–17. 10.1016/j.meatsci.2018.04.008 29684844

[2] Sharma Thind SS, Kaur A. In vitro meat production system: why and how? J Food Sci Technol. 2017; 52 (12): 7599–7607. 10.1007/s13197-015-1972-3 26604337 PMC4648904

[3] Siegrist M, Sutterlin B, Hartmann C. Perceived naturalness and evoked disgust influence acceptance of cultured meat. Meat Sci. 2018; 139: 213–219. 10.1016/j.meatsci.2018.02.007 29459297

[4] Bryant C, Szejda K, Parekh N, Desphande V, Tse B. A Survey of Consumer Perceptions of Plant-Based and Clean Meat in the USA, India, and China. Frontiers in Sustainable Food Systems. 2019; 3: 11. 10.3389/fsufs.2019.00011

[5] Mancini MC, Antonioli F. Exploring consumers’ attitude towards cultured meat in Italy. Meat Sci. 2019; 150: 101–110. 10.1016/j.meatsci.2018.12.014 30616073

[6] Gómez-Luciano CA, de Aguiar LK, Vriesekoop F, Urbano B. Consumers’ willingness to purchase three alternatives to meat proteins in the United Kingdom, Spain, Brazil and the Dominican Republic. Food Quality and Preference. 2019; 78: 103732. 10.1016/j.foodqual.2019.103732

[7] Valente JPS, Fiedler RA, Sucha Heidemann M, Molento CFM. First glimpse on attitudes of highly educated consumers towards cell-based meat and related issues in Brazil. PLoS ONE. 2019; 14(8): e0221129. 10.1371/journal.pone.0221129 31469862 PMC6716657

[8] Wilks M, Phillips CJ. Attitudes to in vitro meat: A survey of potential consumers in the United States. PLoS ONE. 2017; 12(2): e0171904. 10.1371/journal.pone.0171904 28207878 PMC5312878

[9] Wilks M, Phillips CJC, Fielding K, Hornsey M J. Testing potential psychological predictors of attitudes towards cultured meat. Appetite. 2019; 136: 137–145. 10.1016/j.appet.2019.01.027 30731104

[10] Weinrich R, Strack M, Neugebauer F. Consumer acceptance of cultured meat in Germany. Meat Sci. 2020; 162: 107924. 10.1016/j.meatsci.2019.107924 31732401

[11] Verbeke W, Marcu A, Rutsaert P, Gaspar R, Seibt B, Fletcher D, et al. ’Would you eat cultured meat?’: Consumers’ reactions and attitude formation in Belgium, Portugal and the United Kingdom. Meat Sci. 2015; 102: 49–58. 10.1016/j.meatsci.2014.11.013 25541372

[12] Bekker GA, Fischer ARH, Tobi H, van Trijp HCM. Explicit and implicit attitude toward an emerging food technology: The case of cultured meat. Appetite. 2017; 108: 245–254. 10.1016/j.appet.2016.10.002 27717657

[13] Slade P. If you build it, will they eat it? Consumer preferences for plant-based and cultured meat burgers. Appetite. 2018; 125: 428–437. 10.1016/j.appet.2018.02.030 29501683

[14] Bryant CJ, Barnett JC. What’s in a name? Consumer perceptions of in vitro meat under different names. Appetite. 2019; 137: 104–113. 10.1016/j.appet.2019.02.021 30840874

[15] Cohen E, Cohen E. Applying best‐worst scaling to wine marketing. International Journal of Wine Business Research. 2009; 21(1): 8–23. 10.1108/17511060910948008

[16] Erdem S, Rigby D, Wossink A. Using best–worst scaling to explore perceptions of relative responsibility for ensuring food safety. Food Policy. 2012; 37(6): 661–670. 10.1016/j.foodpol.2012.07.010

[17] Sawtooth Software. The MaxDiff System. Technical Paper 2020. Sawtooth Software, Provo, Utah.

[18] Grunert KG. Food quality and safety: consumer perception and demand. European Review of Agricultural Economics. 2005; 32(3): 369–391. 10.1093/eurrag/jbi011

[19] Laestadius LI, Caldwell MA. Is the future of meat palatable? Perceptions of in vitro meat as evidenced by online news comments. Public Health Nutr. 2015; 18(13): 2457–2467. 10.1017/S1368980015000622 25818555 PMC10271528

[20] Bekker GA, Tobi H, Fischer ARH. Meet meat: An explorative study on meat and cultured meat as seen by Chinese, Ethiopians and Dutch. Appetite. 2017; 114: 82–92. 10.1016/j.appet.2017.03.009 28323057

[21] Rolland NCM, Markus CR, Post MJ. The effect of information content on acceptance of cultured meat in a tasting context. PLoS ONE. 2020; 15(4): e0231176. 10.1371/journal.pone.0231176 32298291 PMC7162467

[22] Zhang M, Li L, Bai J. Consumer acceptance of cultured meat in urban areas of three cities in China. Food Control. 2020; 118: 107390. 10.1016/j.foodcont.2020.107390

[23] Post MJ, Levenberg S., Kaplan DL, Genovese N., Fu J., Bryant CJ, et al. Scientific, sustainability and regulatory challenges of cultured meat. Nature Food. 2020; 1(7): 403–415. 10.1038/s43016-020-0112-z

[24] Mattick CS, Landis AE, Allenby BR, Genovese NJ. Anticipatory life cycle analysis of in vitro biomass cultivation for cultured meat production in the United States. Environ Sci Technol. 2015; 49: 11941–11949. 10.1021/acs.est.5b01614 26383898

[25] Tuomisto HL, de Mattos MJ. Environmental impacts of cultured meat production. Environ Sci Technol. 2011; 45: 6117–6123. 10.1021/es200130u 21682287

[26] Schaefer GO, Savulescu J. The ethics of producing in vitro meat. J Appl Philos. 2014; 31: 188–202. 10.1111/japp.12056 25954058 PMC4419201

[27] IBGE. Instituto Brasileiro de Geografia e Estatística: Sinopse do Censo Demográfico. 2010. https://biblioteca.ibge.gov.br/visualizacao/livros/liv49230.pdf (accessed on 05 January 2020)

