# Supplementary material for: Analyzing the importance of attributes for Brazilian consumers to replace conventional beef with cultured meat
Source: PLoS One. 2021 May 7;16(5):e0251432. doi: 10.1371/journal.pone.0251432 (PMC8104404; doi:10.1371/journal.pone.0251432)
Supplement: S1 Survey — (DOCX) [file pone.0251432.s001.docx]

Questionário em português:

1. Quantos anos você tem?

( ) anos de idade.

2. Qual seu gênero?

- Masculino (1)
- Feminino (2)
- Outro (3) ....................................................

3. Qual é o seu nível mais alto de educação?

- Ensino Fundamental incompleto (1)
- Ensino Fundamental completo (2)
- Ensino Médio incompleto (3)
- Ensino Médio completo (4)
- Ensino Superior incompleto (5)
- Ensino Superior completo (6)
- Mestrado incompleto (7)
- Mestrado completo (8)
- Doutorado incompleto (9)
- Doutorado completo (10)

4. Qual sua renda mensal?

- Sem renda (1)
- Até 1 salário mínimo (R$998,99) (2)
- De 1 a 3 salários mínimos (R$998,99 a R$2.996,97) (3)
- De 3 a 6 salários mínimos (R$2.996,97 a R$5.993,94) (4)
- De 6 a 9 salários mínimos (R$5.993,94 a R$8.990,91) (5)
- Acima de 9 salários mínimos (acima de R$8.990,91) (6)

5. Qual sua ocupação?

- Estudante (1)
- Empregado (2)
- Empreendedor (3)
- Funcionário Público (4)
- Aposentado (5)
- Desempregado (6)
- Dona de casa (7)
- Outro (por favor especifique) (8) .....................................

6. Em qual região do Brasil você mora?

- Sul (1)
- Sudeste (2)
- Centro Oeste (3)
- Nordeste (4)
- Norte (5)

7. Como são seus hábitos alimentares?

- Carnívoro (pessoa que consome carnes em sua alimentação) (1)
- Vegetariano (pessoa que não consome carnes em sua alimentação) (2)
- Vegano (pessoa que não consome nenhum tipo de alimento de origem animal, por ex: carne, ovos, leite) (3)
- Outro (por favor especifique) (4) ..........................................

8. Como é seu consumo semanal de carne bovina?

- Não consome (1)
- De 1-2 vezes (2)
- De 3- 5 vezes (3)
- De 6 -10 vezes (4)
- Acima de 10 vezes (5)

9. Quão natural você acha que será a carne bovina de laboratório quando comparada a carne bovina convencional?

- Muito menos natural (1)
- Menos natural (2)
- Nem mais natural e nem menos natural (3)
- Mais natural (4)
- Muito mais natural (5)

10. Você provaria carne bovina feita em laboratório?

- Sim (1)
- Não (2)

11. Você comeria regularmente carne bovina feita em laboratório?

- Sim (1)
- Não (2)

12. Você substituiria das suas refeições a carne bovina convencional ou proteínas (para vegetarianos e veganos) pela carne bovina feita em laboratório?

- Sim (1)
- Não (2)

Escala melhor-pior

Carne de laboratório é carne produzida a partir de células-tronco retiradas de um animal vivo, por meio de técnicas de engenharia de tecidos. Essas células irão proliferar em um meio rico em nutrientes em um laboratório da indústria alimentícia. Nenhum animal é abatido. Esta poderia ser uma alternativa à carne tradicional como a conhecemos hoje em dia. O produto não deve ser confundido com substitutos de carne como tofu ou quorn porque é carne real. A carne de laboratório deve imitar carne em todas as suas sensações físicas, como aparência visual, cheiro, textura e sabor. Em agosto de 2013, em Londres teve a inauguração (degustação) do primeiro hambúrguer feito em laboratório do mundo.

Imagine que a carne bovina de laboratório esteja comercialmente disponível em supermercados, açougues e restaurantes. Para cada um dos 10 conjuntos a seguir, selecione o motivo mais importante e o menos importante que faria você substituir a carne bovina convencional pela carne bovina de laboratório.

Exemplo de choice set

| MAIS IMPORTANTE  (apenas uma resposta) | ATRIBUTO | MENOS IMPORTANTE  (apenas uma resposta) |
| --- | --- | --- |
|  | Ser mais barata que a carne bovina convencional; |  |
|  | Gerar maior bem-estar animal que o gerado pela carne bovina convencional; |  |
|  | Ser mais popular que a carne bovina convencional; |  |
|  | Ser mais saborosa que a carne bovina convencional; |  |
