# Supplementary material for: Analyzing the importance of attributes for Brazilian consumers to replace conventional beef with cultured meat
Source: PLoS One. 2021 May 7;16(5):e0251432. doi: 10.1371/journal.pone.0251432 (PMC8104404; doi:10.1371/journal.pone.0251432)
Supplement: S2 Survey — (DOCX) [file pone.0251432.s002.docx]

1. How old are you?

( ) years.

2. Gender:

( ) Male (1)

( ) Female (2)

( ) Other (3) ....................................................

3. Educational attainment:

( ) incomplete elementary school (1)

( ) complete elementary school (2)

( ) incomplete high school (3)

( ) complete high school (4)

( ) incomplete bachelor degree (5)

( ) complete bachelor degree (6)

( ) postgraduate studies (7, 8, 9, 10)

4. Income

( ) no income (1)

( ) up to R$998,99 (2)

( ) from R$998,99 to R$2.996,97 (3)

( ) from R$2.996,97 to R$5.993,94 (4)

( ) from R$5.993,94 to R$8.990,91 (5)

( ) above R$8.990,91 (6)

5. Employment:

( ) student (1)

( ) employed (2)

( ) entrepreneur (3)

( ) public officer (4)

( ) retired (5)

( ) unemployed (6)

( ) housewife (7)

( ) Others (8)

6. Living region:

( ) South (1)

( ) Southeast (2)

( ) Center west (3)

( ) Northeast (4)

( ) North (5)

7. Dietary lifestyle

( ) meat consumers (1)

( ) vegetarian (2)

( ) others (3, 4)

8. Weekly beef consumption

( ) No consumption (1)

( ) 1-2 meals (2)

( ) 3- 5 meals (3)

( ) 6-10 meals (4)

( ) more than 10 meals (5)

9. How natural do you perceive cultured meat compared to conventional beef meat?

( ) much less natural (1)

( ) less natural (2)

( ) Neutral (3)

( ) more natural (4)

( ) much more natural (5)

10. Would you try cultured meat?

( ) Yes (1)

( ) No (2)

11. Would you regularly eat cultured meat?

( ) Yes (1)

( ) No (2)

12. Would you replace conventional beef by cultured meat in your meals?

( ) Yes (1)

( ) No (2)

Best Worst Scale

“Cultured meat is meat that is grown from stem cells using tissue-engineering techniques. These cells are then transported to a food industry lab where the cells will proliferate in a nutrient-rich medium. Animals are not killed. This could be an alternative to traditional meat as we know it nowadays. Cultured meat should not be confused with meat substitutes like tofu or quorn, because it is real meat. Currently it is not commercially available, though research is being conducted to introduce it as a potential new meat production technique for the future. The unveiling of the world's first in vitro hamburger occurred in London, August 2013. Now, imagine that cultured meat is available at supermarkets, butcher shops and restaurants. For each of the next ten choice sets, choose the most and the least important attribute that would influence you to replace conventional beef meat with cultured meat”.

Example of choice set

| Most important (one option) | Attributes | Least important (one option) |
| --- | --- | --- |
|  | If cultured meat were cheaper than conventional beef meat. |  |
|  | If cultured meat were more animal friendly than conventional beef meat. |  |
|  | If cultured meat were more popular than conventional beef meat. |  |
|  | If cultured meat were tastier than conventional beef meat. |  |
